# ﻿*Primulamedogensis*, a new species of Primulaceae from Tibet of China

**DOI:** 10.3897/phytokeys.230.107008

**Published:** 2023-08-04

**Authors:** Wen-Bin Ju, Heng-Ning Deng, Feng Liu, Xing-Jin He, Xin-Fen Gao, Bo Xu

**Affiliations:** 1 China-Croatia “Belt and Road” Joint Laboratory on Biodiversity and Ecosystem Services, Key Laboratory of Mountain Ecological Restoration and Bioresource Utilization & Ecological Restoration Biodiversity Conservation, Chengdu Institute of Biology, Chinese Academy of Sciences, Chengdu 610041, Sichuan, China Chengdu Institute of Biology, Chinese Academy of Sciences Chengdu China; 2 Key Laboratory of Bio-Resources and Eco-Environment of Ministry of Education, College of Life Sciences, Sichuan University, Chengdu 610065, Sichuan, China Sichuan University Chengdu China; 3 University of Chinese Academy of Sciences, Beijing 100049, China University of Chinese Academy of Sciences Beijing China; 4 Tibet Autonomous Region Research Institute of Forestry Inventory and Planning, Lhasa 850000, China Tibet Autonomous Region Research Institute of Forestry Inventory and Planning Lhasa China

**Keywords:** Morphological characters, *Primula* sect. *Cordifoliae*, taxonomy

## Abstract

We present a description of a newly discovered species, *Primulamedogensis*, found in southern Xizang, China. Additionally, we explore distinctive morphological characteristics that aid in its taxonomy. The new species belongs to sect. Cordifoliae and exhibits morphological similarities to *P.baileyana* and *P.rotundifolia*. However, it can be distinguished by its densely grayish-haired roots, petioles that are 3–7 times longer than the leaf blades, a short stock surrounded by straight and withered petioles, reniform leaf blades with revolute margins, scapes shorter than or equal to leave and both at flowering and in fruiting, flowers solitary on the scapes.

## ﻿Introduction

The genus *Primula* L. is known for its remarkable complexity within the realm of angiosperm taxonomy. Comprising approximately 500 herbaceous plant species, it predominantly thrives in moderate and cold regions of the Northern Hemisphere; individual species can be found in the mountains of South America and Africa, as well as tropical Asia (Hu 1990, 1994; Hu and Kelso 1996; APG 2016). In China, more than 300 species of the genus are distributed (Richards 2003).

Section Cordifoliae, established by Pax (1889), consists of seven species primarily found in the Eastern Himalayas. These species typically inhabit alpine rocky areas beyond the tree-line on the southern side of the Eastern Himalayas (Richards 2003). The distinguishing characteristics of this section include withered leaves and bud scales at the base, long and slender discrete petioles, and predominantly round leaves with a cordate base.

During botanical explorations in Motuo County, Xizang Province, located in southwest China, we collected a remarkable species of *Primula* that thrives among mosses, often in crevices on wet cliffs and among boulders. After subsequent examination of herbarium specimens available at CDBI, and careful consultation of literature (Pax 1889; Pax and Knuth 1905; Smith and Forrest 1928; Smith and Fletcher 1943; Hara 1966, 1971; Ohashi 1975; Fenderson 1986; Hu 1986, 1990; Naithani 1990; Fang 1994, 2003; Hu and Kelso 1996; Grierson and Long 1999; Wu 1999; Bista et al. 2001; Sun and Zhou 2002; Kress et al. 2003; Richards 2003; Basak et al. 2014; Xu et al. 2014; Rajbhandari et al. 2021; Li et al. 2023), we identified that this population represents a new species of Primulasect.Cordifoliae by its morphological characters, which is similar to *Primulabaileyana* Kingdon-Ward and *P.gambeliana* Watt. In this study, we provide a detailed description of this new species based on observations of living plants in the field and pressed specimens in the herbarium.

## ﻿Materials and methods

The descriptions and photographs provided in this study were derived from an extensive analysis of the habits and characteristics observed in wild populations during field surveys. Specimens of the new species were collected from the designated type locality and have been stored at CDBI. To supplement our examination, we also accessed digital specimens available online through various platforms, including the Chinese Virtual Herbarium (http://www.cvh.ac.cn/), the JSTOR Global Plants (https://plants.jstor.org/), the Global Biodiversity Information Facility (https://www.gbif.org/), and the Europeana (https://www.europeana.eu), especially type specimens from A, BM, E, K, P, US.

## ﻿Taxonomic treatment

### ﻿ Primula medogensis

Taxon classificationPlantaeEricalesPrimulaceae

W.B.Ju, Bo Xu & X.F.Gao sp. nov.

E6473EFC-17B8-50DB-B81B-40D68E99B538

urn:lsid:ipni.org:names:77324820-1

#### Diagnosis.

This new species is similar to *P.baileyana* and *P.rotundifolia*, but it differs from them in having roots with hairs, straight petiole remaining from the previous year, leaf blade reniform and revolute at the margin, petiole more than 3 times the length of the leaf blade, scape equal to or shorter than the leave, flower solitary at the apex of the scape, capsule shorter than the calyx.

#### Type.

China. Xizang: Motuo City, Duoxiongla, growing in moist rock crevices covered with moss. 31°04'N, 103°11'E, elevation ca. 3607 m, 18 May 2021, *W. B. Ju & X. Li* YLZB07293 (holotype CDBI!; isotypes KUN!, PE!).

#### Description.

A perennial plant with a short stock, or up to 2.0 × 1.0 cm, usually girt at the base by the straight and withered petioles. ***Roots*** reddish, tuft of wiry, woody on maturity, covered with grayish hairs. Bud scales usually girt at the base by imbricate ovate-oblong to ovate with pale-yellow farinose on the abaxially surface. ***Leaves*** including the petiole 1.2–12.5 cm long; leaf blade reniform or suborbicular, but mostly reniform, 0.3–1.5 × 0.4–2.3 cm, firm papery or subleathery, glabrous and with potentially farinose glands above, copious pale-yellow farina on the lower surface, margin dentate revolute on the lower surface, with a deeply cordate to occasionally truncate base, lateral veins 3–4 pairs; petioles 0.9–11.0 cm long, reddish brown, glabrous, slightly broadened and membranous towards the base. ***Scape*** 1, sparsely short-stalked glandular, 3.0–10.5 cm tall, erect, hardened in fruiting, usually with a single flower; bracts linear-lanceolate, 0.2–0.5 cm long; pedicels 0.2–1.2 cm long, sparsely short-stalked glandular, not extended in fruit. ***Flowers*** heterostylous. Calyx campanulate, 5–8 mm long, glabrous outside, green, inside of pale-yellow farinose, parted nearly to base; lobes lanceolate, margin entire, apex acute, veins 3 with not prominent. Corolla pinkish-purple with a golden-yellow eye, annulate; limb 15–25 mm across, funnelform; lobes spreading, 6–11 × 5–10 mm, broadly obovate, deeply emarginate. ***Thrum flower***: corolla tubes 10–15 mm in length, 2–3 mm in diameter, 2 times the length of calyx, widely ampliated above insertion of stamens; stamens situated at the near apex of the corolla tube; style 3–4 mm. ***Pin flower***: corolla tubes 9–13 mm in length, 2–3 mm in diameter, 2 times the length of calyx; stamens inserted at the middle of corolla tube, style as long as tube. ***Capsule*** broad-ovoid to globose, shorter than calyx, dehiscence by apical valves. ***Seeds*** numerous, free within capsule at maturity, irregularly ovoid-quadrate or pyriform, ca. 0.5–0.8 mm, brown, testa reticulate.

#### Phenology.

Flowering occurs in May to June; fruiting at the end of June.

#### Etymology.

The specific epithet refers to the administrative county name of the type locality viz Motuo (Medog) county in southeast Xizang Autonomous Region, China.

#### Distribution and habitat.

The new species is currently known only from its type locality in Lage, Motuo (Medog) county, Xizang Autonomous Region. It grows in the cracks of steep wet cliffs covered with moss, at elevations of 3550–3700 m.

#### Additional specimens examined (Paratypes).

China, Xizang Autonomous Region, Motuo County, Duoxiongla, it grows in the cracks of steep wet cliffs covered with moss, at elevations of 3626 m, 31 May 2015, *Bo Xu & X.M.Zhou* YLZB01705 (CDBI!).

#### Conservation status.

Currently, only one population have been found. The size of the Lage population remains unknown. According to the IUCN red list criteria (IUCN 2019), the conservation status of the new species should be better categorized as ‘Data Deficient (DD)’. Further explorations in the adjacent mountainous tracts are necessary for an adequate assessment.

#### Discussion.

Section Cordifoliae is a small group within the genus *Primula* that has undergone multiple revisions. Currently, it includes several species such as *P.baileyana* Kingdon-Ward, *P.caveana* W.W.Smith, *P.gambeliana* Watt, *P.macklinae* A.J.Richards (not validly published), *P.littledalei* I.B.Balfour & Watt, *P.ramzanae* Smith & Fletcher and *P.rotundifolia* Wallich (Richards 2003). These species primarily inhabit the tree-line on mountain rocks of the southern slopes of the Himalayas, at altitudes ranging from 3300 to 6000 meters. The most western species, *P.ramzanae*, is exclusively found in Phoksumdo Lake in western Nepal. The most eastern species in this section is one incompletely known species *P.macklinae*, found in northern Myanmar. *P.littledalei* extends from the southern slopes to Lhasa in the Xizang Autonomous Region, and together with *P.baileyana* is only found in China. The species represented by *P.rotundifolia*, *P.gambeliana*, *P.caveana* are from central Nepal through Sikkim to Bhutan and Tibet. The distribution area of the new species overlaps with *P.baileyana* and *P.littledalei* of this section, which is the lowest elevation distribution except *P.macklinae*.

**Figure 1.**
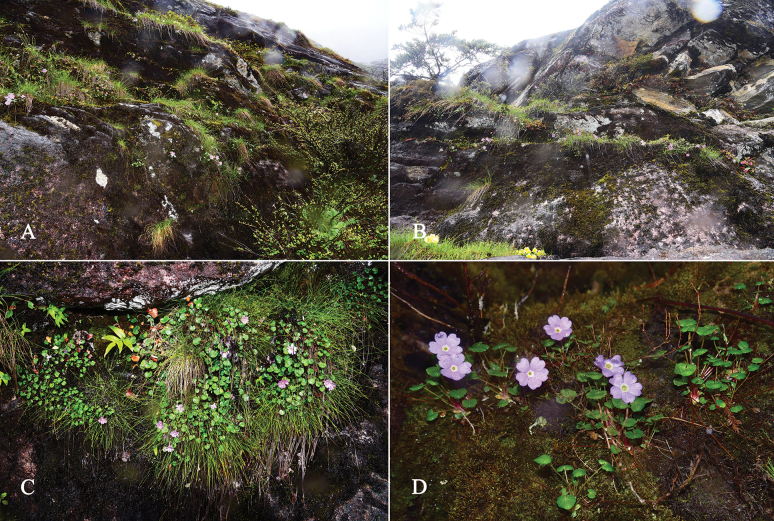
Habitat of the *Primulamedogensis* sp. nov. (**A–D**)

**Figure 2.**
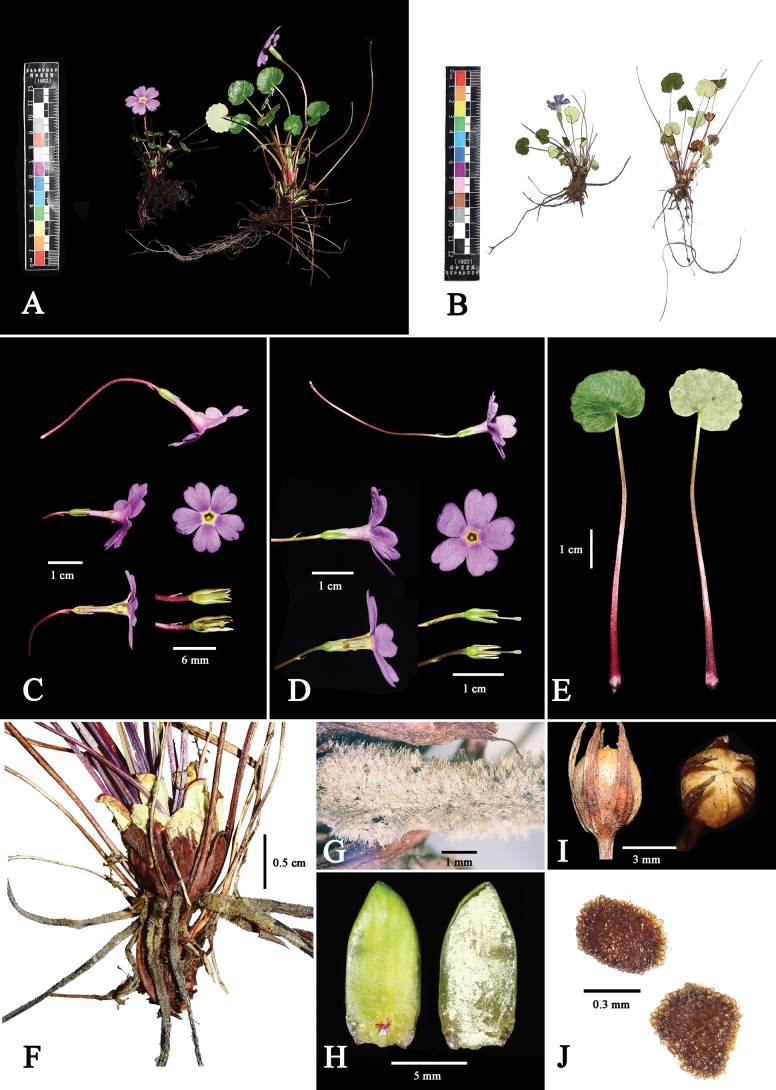
*Primulamedogensis* sp. nov. **A** fresh plants **B** pressed specimen **C** thrum flower **D** pin flower **E** leaves **F** plants base, showing roots, bud scales and petioles **G** roots covered with greyish hairs **H** bud scales **I** capsule **J** seed.

**Figure 3.**
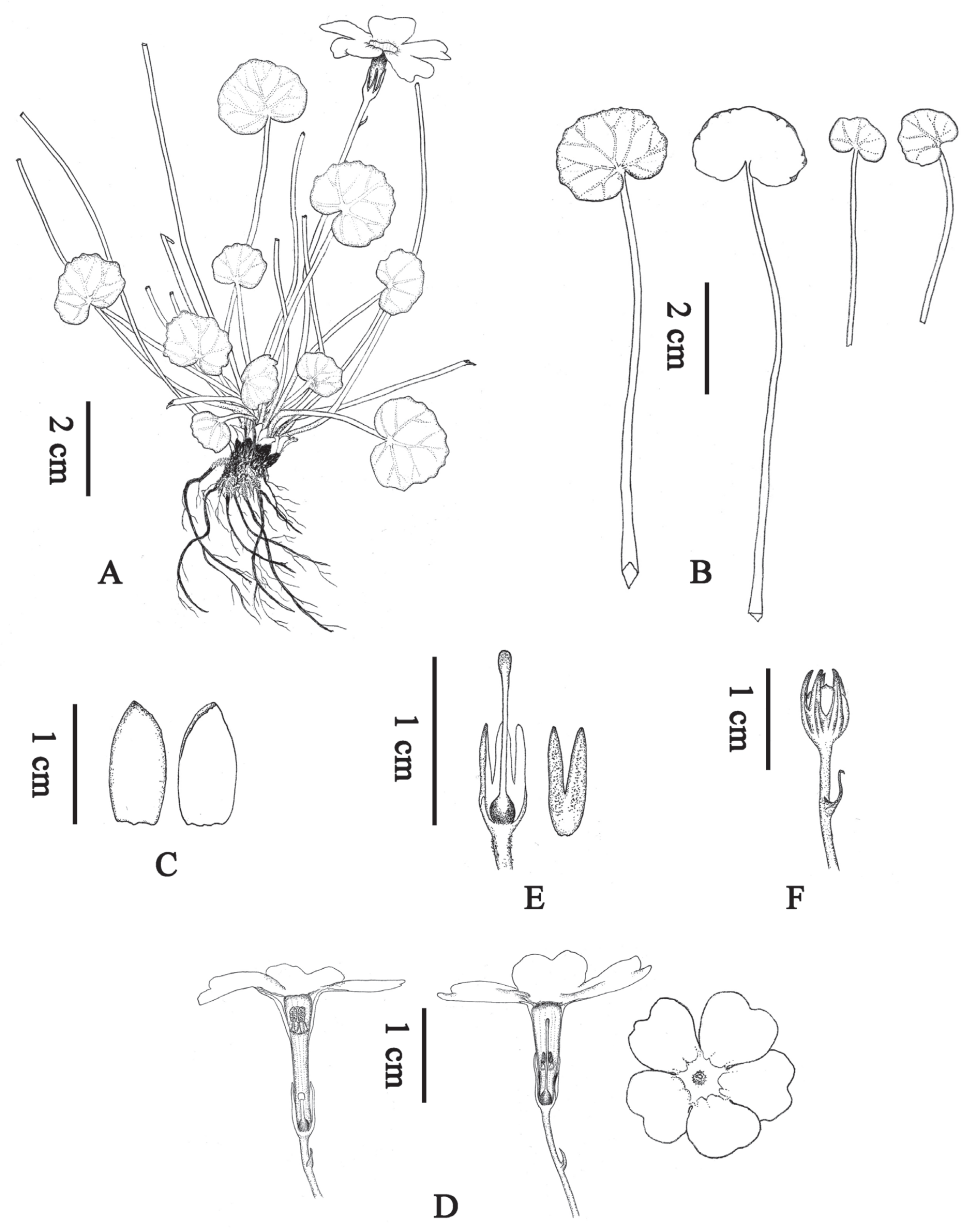
*Primulamedogensis* sp. nov. **A** habit **B** leaves **C** bud scales **D** flowers: thrum flower, pin flower, and front of the flower **E** calyx and ovary **F** capsule.

Morphologically, *P.medogensis* shares certain similarities with *P.baileyana* and *P.rotundifolia*, both of which also belong to P.sect.Cordifoliae. These similarities include mealy, deciduous plants arising from substantial resting buds, the base of the leaf cluster often has many withered petioles, leaf blades are usually reniform or suborbicular, base cordate, with a conspicuous petiole, corolla pinkish-purple, calyx campanulate, with splitting to below middle, capsule horny (Figs 1–3). However, this new species has a series of morphological characters unique to the section that consists of straight old petioles at the base, petioles 3–7 times longer than the leaf blades, leaf blades mostly reniform with revolute margins, single flowers, and scape shorter than or equal to the leaves. In addition to the unique features described above, *P.medogensis* clearly differs from *P.baileyana* by roots clothed in dense grayish hairs, pale- yellow farinose, smaller leaf blades, calyx lobes with 3 veins, and capsules shorter than the calyx; and it differs from *P.rotundifolia* by its smaller leaf blades, corolla lobes deeply emarginate, the capsule broad-ovoid to globose and shorter than the calyx. For a comprehensive overview of the contrasting characteristics between *P.medogensis*, *P.baileyana*, and *P.rotundifolia*, please refer to Table 1.

**Table 1.** Comparison of morphological characters among *Primulamedogensis*, *P.baileyana* and *P.rotundifolia*.

Characters	*Primulamedogensis*	*P.baileyana*	*P.rotundifolia*
Root	dense grayish hairs	Glabrous	Glabrous
Stem	straight old petioles at the base	twisted old leaves at the base	twisted old petioles at the base
Petiole	3–7 times as long as the leaf blade	1–3 times as long as the leaf blade	1–3 times as long as the leaf blade
Leaf blade	0.4–2.3 cm wide	0.5–4.0 cm wide	4–12 cm wide
	mostly reniform	mostly suborbicular	Suborbicular
	abaxially pale-yellow farinose	abaxially white farinose	abaxially yellow farinose
	margin revolute	margin not revolute	margin not revolute
Scape	one flower	umbel 1–7 flowered	umbel 2–16 flowered
	shorter than or equal to leaves	2–3 times as long as leaves	1.5–2 times as long as leaves
Calyx	pale- yellow farinose inside, veins 3	white farinose inside, veins 5	yellow farinose inside, veins 3
Corolla	lobes deeply emarginate	lobes deeply emarginate	lobes margin entire to obscurely crenulate
Capsule	broad-ovoid to globose, shorter than calyx	ovoid to oblong, ca. as long as calyx	oblong, longer than calyx

## Supplementary Material

XML Treatment for Primula medogensis

## ﻿Additional information

### Author ORCIDs

Wen-Bin Ju https://orcid.org/0000-0002-5665-6290

Heng-Ning Deng https://orcid.org/0000-0002-0581-0649

Xing-Jin He https://orcid.org/0000-0003-2064-0112

Xin-Fen Gao https://orcid.org/0000-0002-5703-1639

Bo Xu https://orcid.org/0000-0003-3507-9321

### Data availability

All of the data that support the findings of this study are available in the main text.
